# Production, purification, and quality assessment of borrelial proteins CspZ from *Borrelia burgdorferi* and FhbA from *Borrelia hermsii*

**DOI:** 10.1007/s00253-024-13195-2

**Published:** 2024-07-23

**Authors:** Mickaël Guérin, Marylène Vandevenne, Alain Brans, André Matagne, Rodrigue Marquant, Elise Prost, Stéphane Octave, Bérangère Avalle, Irene Maffucci, Séverine Padiolleau-Lefèvre

**Affiliations:** 1 Unité de Génie Enzymatique et Cellulaire (GEC), CNRS UMR 7025, Université de Technologie de Compiègne, Compiègne, 60203 France; 2 Laboratory of Enzymology and Protein Folding, InBioS Research Unit, University of Liège, Building B6, Quartier Agora, Allée du 6 Août, 13, Sart-Tilman, Liège, 4000 Belgium; 3 Robotein®, InBioS Research Unit, University of Liège, Building B6, Quartier Agora, Allée du 6 Août, 13, Sart-Tilman, Liège, 4000 Belgium; 4 Protein Factory, InBioS Research Unit, University of Liège, Building B6, Quartier Agora, Allée du 6 Août, 13, Sart-Tilman, Liège, 4000 Belgium; 5 Centre for Protein Engineering, InBioS Research Unit, University of Liège, Building B6, Quartier Agora, Allée du 6 Août, 13, Liège, Sart- Tilman), 4000 Belgium

**Keywords:** CspZ, FhbA, Surface plasmon resonance, Factor H, Factor-H like 1, Protein quality control

## Abstract

**Abstract:**

*Borrelia*, spirochetes transmitted by ticks, are the etiological agents of numerous multisystemic diseases, such as Lyme borreliosis (LB) and tick-borne relapsing fever (TBRF). This study focuses on two surface proteins from two *Borrelia* subspecies involved in these diseases: CspZ, expressed by *Borrelia burgdorferi* sensu stricto (also named BbCRASP-2 for complement regulator-acquiring surface protein 2), and the factor H binding A (FhbA), expressed by *Borrelia hermsii.* Numerous subspecies of *Borrelia*, including these latter, are able to evade the immune defenses of a variety of potential vertebrate hosts in a number of ways. In this context, previous data suggested that both surface proteins play a role in the immune evasion of both *Borrelia* subspecies by interacting with key regulators of the alternative pathway of the human complement system, factor H (FH) and FH-like protein 1 (FHL-1). The recombinant proteins, CspZ and FhbA, were expressed in *Escherichia coli* and purified by one-step metal-affinity chromatography, with yields of 15 and 20 mg or pure protein for 1 L of cultured bacteria, respectively. The purity was evaluated by SDS-PAGE and HPLC and is close to about 95%. The mass of CspZ and FhbA was checked by mass spectrometry (MS). Proper folding of CspZ and FhbA was confirmed by circular dichroism (CD), and their biological activity, namely their interaction with purified FH from human serum (recombinant FH_15-20_ and recombinant FHL-1), was characterized by SPR. Such a study provides the basis for the biochemical characterization of the studied proteins and their biomolecular interactions which is a necessary prerequisite for the development of new approaches to improve the current diagnosis of LB and TBRF.

**Key points:**

•* DLS, CD, SEC-MALS, NMR, HPLC, and MS are tools for protein quality assessment*

•* Borrelia spp. possesses immune evasion mechanisms, including human host complement*

•* CspZ and FhbA interact with high affinity (pM to nM) to human FH and rFHL-1*

**Graphical Abstract:**

**Figure:**
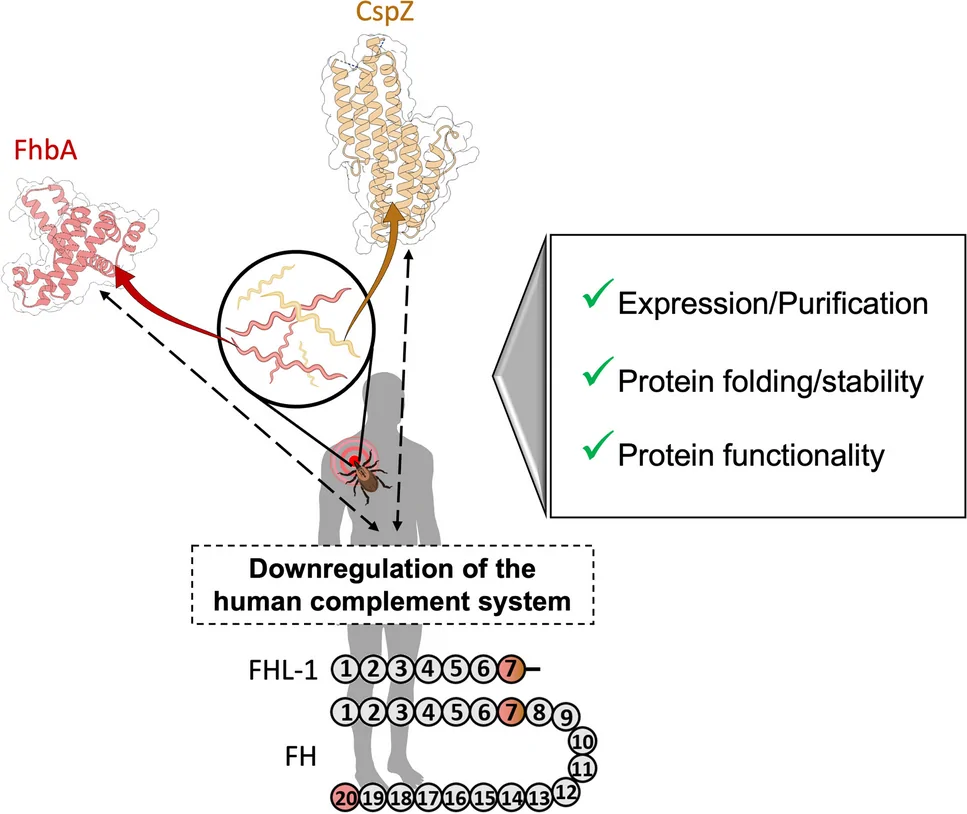

## Introduction

*Borrelia* is a bacterial genus of the spirochete phylum. It comprises three distinct groups, namely (i) the Lyme group (LG) that causes Lyme borreliosis (LB), (ii) the relapsing fever group (TBRF), the etiological agent of relapsing fever (RF), and (iii) the Echidna-Reptile group (REPG). As the latter is non-pathogenic to humans, our study will solely focus on the LG and TBRF groups.

LB, caused by the *B*. *burgdorferi* sensu lato (*Bb*sl) complex, is the most common tick-borne disease in the world, with an estimation of 700,000 cases per year in the USA and Europe. LB presents a variety of symptoms influenced by the infecting *Borrelia* species within the *Bb*sl complex: the characteristic erythema migrans rash and others with less distinctive clinical features, such as fatigue, headache, arthralgia, and myalgia (Guérin et al. 2023). RF, which mainly affects people in America, Asia, and Africa, is caused by a range of subspecies with different geographical distributions (Faccini-Martínez et al. 2022). RF is characterized by a cyclic pattern of high-level spirochetemias and recurrent episodes of fever (Trevisan et al. 2021b). Thus, all *Borrelia* subspecies differ in clinical manifestations, vertebrate host preferences, dissemination within the human body, and also in genomes/antigen expression (Trevisan et al. 2021a, b).

Previous reports have shown that the *Borrelia* genus uses the recruitment of host complement regulators as a tactic to evade the immune response (Kraiczy et al. 2008a, b; Lin et al. 2020; Anderson and Brissette 2021; Kogan et al. 2022).

This study focuses on two different proteins from *B. burgdorferi* sensu stricto (NCBI: txid139) and *B. hermsii* (NCBI: txid140), namely CspZ (BbCRASP-2/BBH06) and FhbA, respectively. These proteins are known for their interaction with the factor H (FH) and the H-factor-like protein 1 (FHL-1), which are two regulators of the complement system (Hovis et al. 2004; Kraiczy et al. 2008a; Meri et al. 2013; Kogan et al. 2022). On one hand, FH is a serum glycoprotein composed of 20 globular short consensus repeats (SCRs) and is constitutively expressed by the liver (Adinolfi et al. 1981; Schwaeble et al. 1987) and some other cell types (Brooimans et al. 1990; Parente et al. 2017). Serum FH concentrations range from 200 to 800 µg/mL, depending on genetic and environmental factors (Esparza-Gordillo et al. 2004; Clark et al. 2014; Parente et al. 2017). Given the significant challenges associated with recombinant production of full-length FH, studies have employed truncated forms. These truncated forms are designated as FH_X-Y_, indicating the X to Y SCR domains involved. On the other hand, FHL-1 is a truncated form of FH that results from alternative splicing of the CFH gene. It shares the first seven domains (or SCRs) with FH but has a unique 4-amino acid C-terminal end (-SFTL) (Schwaeble et al. 1987). Although data on FHL-1 levels are limited, some studies reported a concentration of approximately 30 to 50 µg/mL in human plasma (Zipfel and Skerka 1999; Friese et al. 2000). Upon bacterial entry into the human bloodstream, the complement system can be activated by the alternative pathway (AP). Briefly, the AP is activated by the binding of C3b protein to the bacterial surface, which triggers the complement amplification (Trouw and Daha 2011), leading to the formation of C3 convertase and subsequently C5 convertase. This ultimately results in the production of the bacteriolytic pore-forming membrane attack complexes (MACs) (Merle et al. 2015a, b). FH and FHL-1 play a crucial role in the AP activation of the human complement system by preventing excessive activation of the AP and self-directed damage to host cells.

Spirochetes are vulnerable to complement-mediated lysis, but they have developed strategies to evade the host immune system (Anderson and Brissette 2021). *Bb*sl and other microorganisms that belong to genus *Candida* (Valand et al. 2022) and to genus *Leptospira* (Barbosa and Isaac 2020) have been shown to use a strategy where they interact with the host’s FH and FHL-1 to escape the human complement system and disseminate within the host. CspZ interacts with FH via domains 5 to 7, and FhbA interacts with FH either via domains 5 to 7 or domains 19 to 20 (Hovis et al. 2004; Kraiczy et al. 2008a; Meri et al. 2013; Kogan et al. 2022). CspZ and FhbA have been shown to bind FHL-1 via a conformational epitope (Hartmann et al. 2006; Hovis et al. 2006; Rogers and Marconi 2007; Kraiczy et al. 2008a, b; Brangulis et al. 2014). The binding of FH and FHL-1 with bacterial surface proteins of *Borrelia* results in the inactivation of C3b, facilitating the escape of the human complement system and dissemination of *Borrelia* within the host.

FhbA is a 20.5 kDa surface-exposed lipoprotein encoded by a single genetic locus located on the linear plasmid lp220 (Hovis et al. 2004). It comprises nine α-helices and exhibits high-affinity interactions with FH (*K*_*D*_ = 30 nM as assessed by microscale thermophoresis (MST) (Kogan et al. 2022). The *K*_*D*_ of FhbA for recombinant (*r*) FH_19-20_ was estimated at 82 ± 20 nM using MST. However, no further data are available on the interaction with FHL-1 (Kogan et al. 2022). Studies have shown that FhbA interaction with FH reduces the complement activation and enhances the bacterial serum survival. Therefore, FhbA plays a crucial role in the survival of bacteria in the bloodstream during the spirochetemic phase of RF infection, where bacterial concentration reaches 10^5^-10^6^ bacteria/mL. FhbA is an interesting protein that may be useful for diagnosis as FhbA-like genes are conserved in the majority of the RF clade (Kogan et al. 2022).

CspZ is a lipoprotein located on the surface of *B. burgdorferi*. This protein is highly conserved both between and within different Lyme borreliae species (> 85% amino acid identity between species and 98% between *Bb* strains) (Marcinkiewicz et al. 2023) and is encoded by the *cspZ* gene located on the linear plasmids lp28-3. In established mammalian infections, CspZ is predominantly produced. However, in vitro cultures lead to a significant reduction of its expression at the surface of the cells (Bykowski et al. 2007). The expression level of the *cspZ* gene is influenced by various parameters, including temperature and pH. This suggests that CspZ is highly sensitive to environmental cues and is tailored to the specific conditions encountered during mammalian infection (Bykowski et al. 2007). In addition, recent studies shown that CspZ interacts strongly with recombinant human FH_6-7_, with binding constants ranging from 150 to 300 nM assessed by ELISA assays and SPR, respectively (Marcinkiewicz et al. 2019, 2023). Although western blot (WB) analysis has provided evidence of CspZ binding to human FH or FHL-1 (Hartmann et al. 2006), quantitative data on the interaction strength is not available. In the same study, researchers presented compelling evidence for the crucial role of CspZ in *Borrelia burgdorferi*’s ability to avoid complement-mediated lysis and, therefore, to establish systemic infection in vertebrate hosts. Furthermore, a *cspZ*-deficient mutant and a strain expressing a nonbinding-FH CspZ variant exhibited an impaired ability to cause bacteraemia and colonize tissues in mice or quail. However, the virulence of these mutants was restored in complement C3-deficient mice (Marcinkiewicz et al. 2019).

The abovementioned studies have provided insights into the relationship between human complement proteins and bacterial proteins of the *Borrelia* genus. They contain important information concerning the molecular mechanisms underlying the ability of *Borrelia* to persist and cause disease in mammalian hosts. Furthermore, some data suggest the potential of CspZ and FhbA as valuable diagnosis or therapeutic tools (Marcinkiewicz et al. 2020; Kogan et al. 2022). In this study, the biochemical foundations to characterize these two proteins and their interactions with the human complement proteins (i.e., human purified FH, rFHL-1, and rFH_15-20_) were established with the aim of enabling the development of new detection methods to improve the diagnosis of LB and TBRF.

## Materials and methods

### Materials and instruments

All chemicals for preparing buffers, solutions, and materials were purchased from VWR International (Germany), Merck (Germany), Thermo Fisher (USA), or Carlo Erba Reagents (Italy). BCA measurements were performed with a VANTAstar® microplate reader from BMG Labtech (Germany). Sodium dodecyl-sulfate–polyacrylamide gel electrophoresis (SDS-PAGE) and WB images were taken with the ChemiDoc™ imaging system (Bio-Rad, USA). SPR measurements were performed with a T100 Biacore SPR system (Cytiva), and purification of the protein was achieved using an ÄKTA™ start. SPR sensor chips (CM5) and HiTrap TALON crude column were purchased from Cytiva.

FH and rFHL1 proteins were generously provided by Prof. Clark. Purified human complement FH (reference C5813 from Sigma) comprises 20 SCR domains (NCBI gene number: 3075). rFHL-1, encompassing the first 7 SCRs of FH, is appended with a 4-amino acid C-terminal tail (-SFTL). This protein is fused with a polyhistidine tag at the N-terminus. rFH_15 − 20_ (reference 10,714-H08H) was obtained from Sino Biologicals (Beijing, China). This protein contains the last 5 SCRs of FH (NP_000177.2), corresponding to amino acid residues (Ser 860-Arg 1231), with a polyhistidine tag at the C-terminus and a signal peptide at the N-terminus.

### Production and purification of CspZ

Three plasmids containing *cspZ* were used. Two plasmids were kindly provided by Prof. Marconi (USA) and Dr. Brangulis (Latvia), coding for CspZ with a N-terminal hexahistidine tag, respectively, pET-45(+) and pETM-11. Another plasmid, containing the gene encoding for FhbA (pET-32 Ek-LIC), was kindly provided by Dr. Kogan (Finland). Each plasmid was transformed into *Escherichia coli* BL21 (DE3)*-*competent cells by heat shock at 42 °C. *E. coli* BL21(DE3) cells harboring the recombinant plasmids of interest were grown at 37 °C with shaking (250 rpm) in the Luria-Bertani broth supplemented with 50 µg/mL ampicillin. Protein expression was induced by addition of 1 mM isopropyl-β-d-thiogalactopyranoside (IPTG) for 3 h, and the cells were harvested by centrifugation (6000 × g, 10 min) and lysed using B-PER™ Complete Bacterial Protein Extraction Reagent (reference 78,243, Thermo Fisher) supplemented with a cOmplete™ ULTRA EDTA-free protease inhibitor tablet (reference 5,892,953,001, Roche Diagnostics). The recombinant proteins were purified from clarified cell lysate by Co-NTA affinity chromatography using 1 mL HiTrap® TALON® crude column on ÄKTA™ start protein purification system. Unbound proteins were washed from the resin with PBS (132 mM NaCl; 5 mM Na_2_HPO_4_, 1.2 mM KH_2_PO_4_, pH 7.6). The elution step was set at 90 mM imidazole during 6 min at a flow rate of 1mL/min for all proteins, except for CspZ expressed in pET-45(+). This later was eluted with 30 mM imidazole. After two dialysis—the first one conducted at 4 h at room temperature (RT), followed by the second one O/N at 4 °C in 2 L PBS using 3.5 kDa MWCO dialysis bag (reference 88,242, Thermo Fisher), the concentration of the proteins was measured by Pierce™ BCA Protein Assay Kits (Thermo Fisher). 6X Laemmli buffer was added to 40 µg of crude extract and 10 µg of each purified and dialyzed CspZ and FhbA. The samples were then heated at 95 °C for 5 min. Subsequently, protein identification was performed by SDS-PAGE (12%) followed WB analysis. Migration was carried out at 200 V for 45 min in the migration buffer (25 mM Tris, 192 mM Glycine, 3.5 mM SDS). The proteins were then transferred to the membrane at 100 V for 1 h in transfer buffer (25 mM Tris, 192 mM Glycine, 20% ethanol (vol/vol)) and at 4 °C. Nitrocellulose membranes (reference 10,600,004, Cytiva) were first blocked for 1 h at RT with 5% (w/v) skim milk (reference 70,166, Sigma) in PBS. Membranes were then incubated for 1 h at RT with His-probe (H-3) HRP-conjugated antibody (reference sc-8036-HRP, Santa Cruz Biotechnology) diluted 200-fold in PBS. Three washes with PBS for 5 min with agitation were performed between each step. Revelation was performed using Western Pierce™ ECL Substrate (reference 32,106, Thermo Fisher). SDS-PAGE and WB pictures were taken with the ChemiDoc™ imaging system (Bio-Rad Laboratories, USA). The purification was checked by high-performance liquid chromatography (HPLC). The variable wavelength UV detector was set at a wavelength of 220 nm. The analyte injection volume was 5 µL, and the column temperature was set at 25 °C. An UltiMate 3000 HPLC from Thermo Fisher with a Fortis Bio C18, 3 μm, 4.6 × 100 mm reversed-phase column was used. Two mobile phases, (1) ultrapure water with 0.1 vol % trifluoroacetic acid (TFA) and (2) acetonitrile (ACN) with 0.1 vol % TFA, were used for the linear gradient elution between 5 and 100% ACN in 10 min. The solvent flow rate was set at 1 mL/min. Quantification was performed using external calibration peak area measurement.

Liquid chromatography-high-resolution mass spectrometry (LC-HRMS) was performed using an Agilent 1290 HPLC system with a diode array detector (DAD) and an Agilent Q-TOF 6538 mass spectrometer. HPLC separation was performed on an ACE C4 column (150 × 4.6 mm, 5 μm, 300 A) connected to an Agilent Infinity 1290 HPLC system maintained at 40 °C. The mobile phase consisted of (a) 0.1% formic acid in H_2_O and (b) ACN. The gradient elution program began by maintaining 5% B for 2 min, ramping to 30% B at 10 min, followed by a linear increase to 95% B at 25 min. The mobile phase composition was maintained at 95% B for 5 min, before returning to initial conditions and holding for 4 min. The flow rate was set to 1.200 mL/min. All compounds were detected in positive electrospray ionization (ESI+) mode and calibrated externally. The ESI source was operated with a gas temperature of 350 °C, a voltage of + 3500 V, a drying gas flow rate of 10 L/min, and a nebulizer pressure of 30 psig. The fragmentor voltage was set to 200 V. HRMS spectra were acquired at 2 Hz in the mass range of 100 to 3000 m/z using internal calibration. Data processing was performed using MassHunter B07 software for mass spectrum extraction and Unidec 6.0 software (https://github.com/michaelmarty/UniDec/releases) for deconvoluting the mass spectra to determine the protein mass from multi-charged ions (Marty et al. 2015).

### Nuclear magnetic resonance (NMR)

NMR experiments were performed on a 400 MHz spectrometer (Bruker Avance III HD) equipped with a nitrogen-cooled 5 mm prodigy probe head BBO H&F. The standard pulse sequence “zgesgp” from the Bruker library was used for water signal suppression using a shaped pulse (sinc1.1000) lasting 2 ms. All spectra were recorded at 25 °C with an acquisition time of 4.1 s, a relaxation delay of 1.5 s, and 1024 scans. Data were processed using the Bruker topspin 4.3.0 software. Solutions were prepared by mixing 135 µL of 110 µM protein solution with 15 µL D2O and transferred to 2 mm stem NMR microtubes.

### Circular dichroism (CD)

Far-UV CD spectra (195–260 nm) of CspZ and FhbA were recorded with a Jasco J-810 spectropolarimeter at 20 °C in PBS, using a 1 mm pathlength quartz Suprasil cell (Hellma), with protein concentrations of 0.1 mg/mL. Four consecutive scans (20 nm/min, 1 nm bandwidth, 0.1 nm data pitch, and 2 s digital integration time) were averaged, baselines were subtracted, and no smoothing was applied. Data obtained with an applied high-tension voltage above 600 V were not considered. Data are presented as the molar residue ellipticity ([Ɵ]_MRW_) calculated using the protein molar concentration and the number of residues.

Secondary structure analyses using the CDSSTR (Johnson 1999; Sreerama and Woody 2000), CONTINLL (Provencher and Glöckner 1981; van Stokkum et al. 1990; Sreerama and Woody 2000), and SELCON3 (Sreerama and Woody 1993; Sreerama et al. 1999) algorithms were performed on the CD data with the CDPro software package (Sreerama and Woody 2000), using reference dataset SMP56. The results from the three algorithms were averaged, and the standard deviations between the calculated secondary structures are given in the Results section.

### Dynamic light scattering (DLS) and size exclusion chromatography coupled with multi-angle light scattering (SEC-MALS)

DLS measurements were performed at 20 °C using a DynaPro NanoStar™ (Wyatt Technologies, 10–100 mW He-Ne laser, *λ*_0_ = 658 nm, scattering angle (θ) = 90°). Disposable plastic cuvettes (Eppendorf UVette® 200–1600 nm) were filled with 100 µL of protein sample solution using a protein concentration of 1 mg/mL. Prior to the analysis, the samples were centrifuged for 10 min at 9000 × g to eliminate air bubbles, dust, or other undesired high molecular weight particles. Twenty successive DLS acquisitions were performed per sample. The data acquisition and analysis were performed using the Dynamics software version 7.8.2.18. The hydrodynamic radius (*R*_*H*_) of the different particles in solution was determined based on autocorrelation functions that were adjusted using a regularization (size distribution) fit for multimodal samples. Size distributions using the percentage of intensity rather than the percentage of mass or number is presented.

The protein solutions were analyzed using a SEC-MALS/UV/RI light scattering system that combines a HPLC from Shimadzu (Tokyo, Japan), mini-DAWN TREOS II, and refractometer (Shimadzu), all from Wyatt Technology (Santa Barbara, CA, USA). For each sample, 100 µL at approximately 3.75 mg/mL (for CspZ) or 2.50 mg/mL (for FhbA) was injected onto a Superdex 200 increase 10/300 GL column (Cytiva) in PBS at a flow rate of 0.7 mL/min. An average refractive index increment (dn/dc) of 0.183 mL/g at 658 nm was used for protein concentration determination. The light scattering detector, equipped with a high gain, high dynamic range photodiodes at 3 detection angles, was calibrated with toluene. Data acquisition and analysis were performed using ASTRA 7.3.0 software (Wyatt Technology).

### Surface plasmon resonance (SPR) assays

Protein-protein interactions were analyzed by surface plasmon resonance technique using a Biacore T100 instrument (Cytiva). Carboxylated dextran chips (sensor chip CM5, research-grade from Biacore AB) were used in all the assays and were activated using 0.1 M of N-hydroxyl succinimide and 0.4 M of N-ethyl-N’-(dimethylaminopropyl)-carbodiimide (NHS-EDC) following the Biacore wizard. Subsequently, protein rFHL-1 and purified FH from human plasma (reference C5813, batch number: SLCP3577, Merck), both kindly provided by Prof. Simon Clark, were resuspended in 10 mM acetate buffer, pH 5 at a concentration of 10 µg/mL. Recombinant FH_15 − 20_ was resuspended in the same conditions. After the coupling, free-reacting groups were inactivated using 1 M ethanolamine-HCl (pH 8.5). The amine couplings were performed using PBS supplemented with 0.05% Tween20 as the flow buffer throughout.

Briefly, rFHL-1, rFH_15 − 20_, and FH were used as ligands by coupling via the standard amine-coupling procedure until targeted levels of 200 resonance units (RU) for rFHL-1 and rFH_15 − 20_ and 750 RU for FH were reached. A control flow cell was prepared similarly but without injecting any protein. *Borrelia* recombinant proteins CspZ and FhbA, used as analytes, were dialyzed against running buffer. Kinetic measurements were performed by injecting several amounts of *Borrelia* proteins, ranging from 1 nM to 22 µM for FhbA and from 0.5 nM to 10 µM for CspZ at flow rate of 30 µl/min at 25 °C. Association time was applied for 120 s, followed by a dissociation time of 20 min before regeneration. Surfaces were regenerated by injection of 60 µl of 10 mM Glycine HCl pH 3. The blank run was subtracted from each sensorgram, prior to data processing using the BIAcore T100 evaluation software. Various analyses using single-cycle and multiple-cycle kinetics have been performed. After the procedure, a logarithmic Langmuir 1:1 binding model and the simultaneous ka/kd fitting of the BIA evaluation software were applied (Biacore, AB).

## Results

### Validation of the expression and purification of CspZ and FhbA in *E. coli* BL21(DE3)

**Fig. 1:**
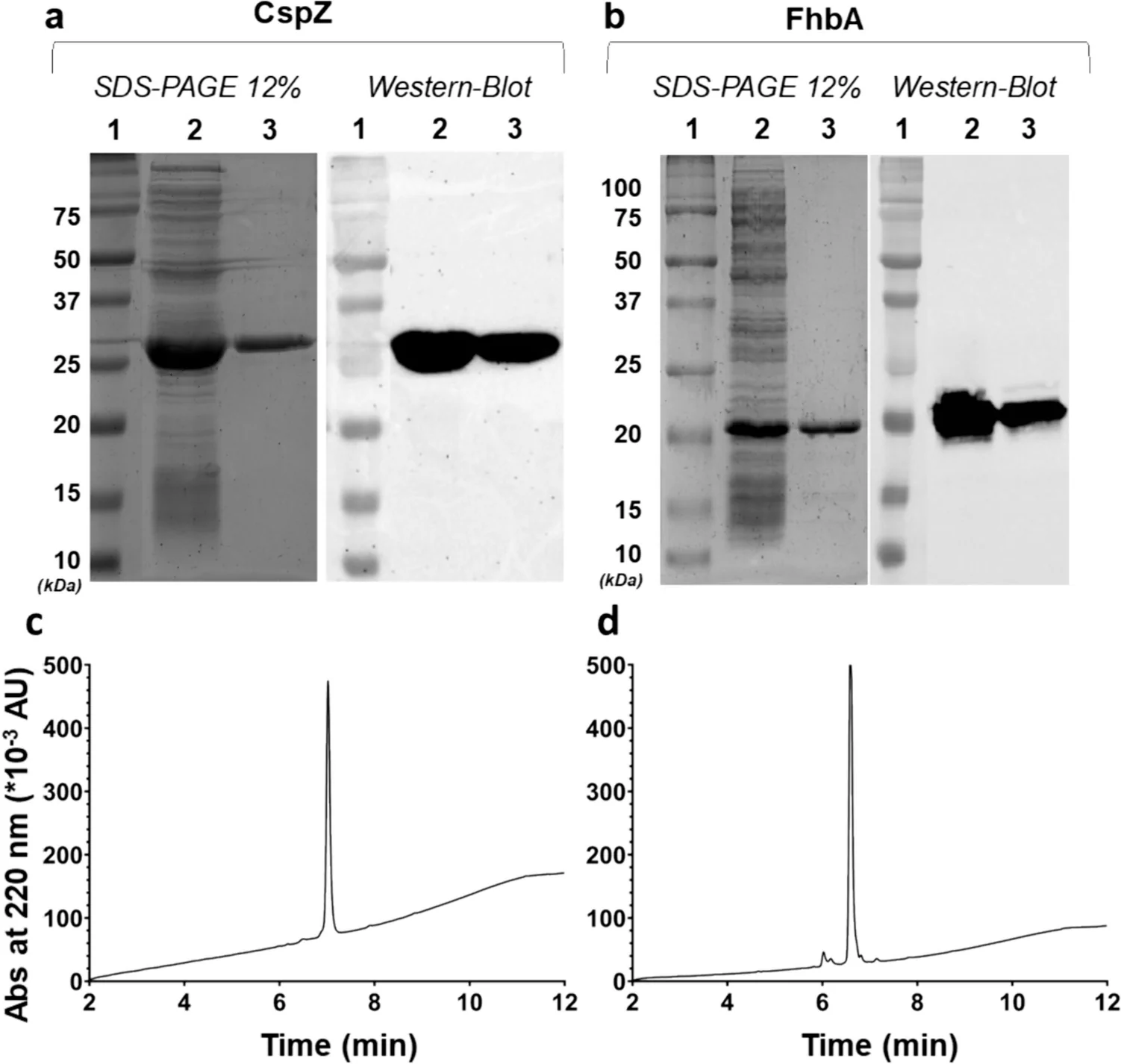
Protein expression and purification visualization on SDS-PAGE gels, Western Blot and HPLC. **a** Migration patterns of soluble extract (lane 2) and purified and dialyzed CspZ protein (lane 3), analyzed on a 12% SDS-PAGE gel stained with Coomassie blue (left) and by western blot detected using horseradish peroxidase (HRP)-conjugated anti-histidine tag antibody (right). **b **Migration patterns of soluble extract (lane 2) and purified FhbA protein (lane 3) were analyzed using a 12% SDS-PAGE gel stained with Coomassie blue (left) and a western blot detected using HRP-conjugated anti-histidine tag antibody (right). **c** HPLC chromatogram of purified CspZ (main peak). **d** HPLC chromatogram of purified FhbA (main peak)

CspZ and FhbA were expressed in *E. coli* BL21(DE3) strain and purified using immobilized metal ion affinity chromatography, as described in the Materials and Methods section. The quality of the purified proteins was assessed according to the best practice recommendation established by the ARBRE-MOBIEU and P4EU networks (Berrow et al. 2021; de Marco et al. 2021). Thus, the crude extract and purified proteins were analyzed using the Coomassie-stained SDS-PAGE, which revealed a prominent band at approximately 27 kDa for CspZ and 22 kDa for FhbA. The apparent molecular masses are in good agreement with the theoretical values (i.e., 27.5 and 23.5, respectively) predicted by ProtParam (https://web.expasy.org/protparam/) analysis (Wilkins et al. 1999). An additional protein identity control was performed by confirming the presence of the N-terminal histidine tags, at the same size as the protein bands (Fig. 1a, b). The overall quantities for CspZ and FhbA were 15 mg and 20 mg of purified protein per liter of culture, respectively.

The purity of the protein and the presence of any impurities were confirmed using high-performance liquid chromatography (HPLC) analysis. The experimental data are illustrated in Fig. 1c, d.

Mass spectrometry confirmed the successful expression of both proteins. Indeed, molecular masses of 27,397 kDa and 23,339 kDa were determined for CspZ and FhbA, respectively, after deconvolution using Unidec software. A dimerized protein with a peak at 46,679 kDa was also observed for FhbA. The observed molecular masses aligned precisely with the predicted values, excluding the N-terminal methionine residues, which confirms the successful expression of both proteins without truncation artifacts (Ben-Bassat et al. 1987; Wingfield 2017).

### Dynamic light scattering (DLS) and size exclusion chromatography–multi-angle light scattering (SEC-MALS) analysis

**Fig. 2:**
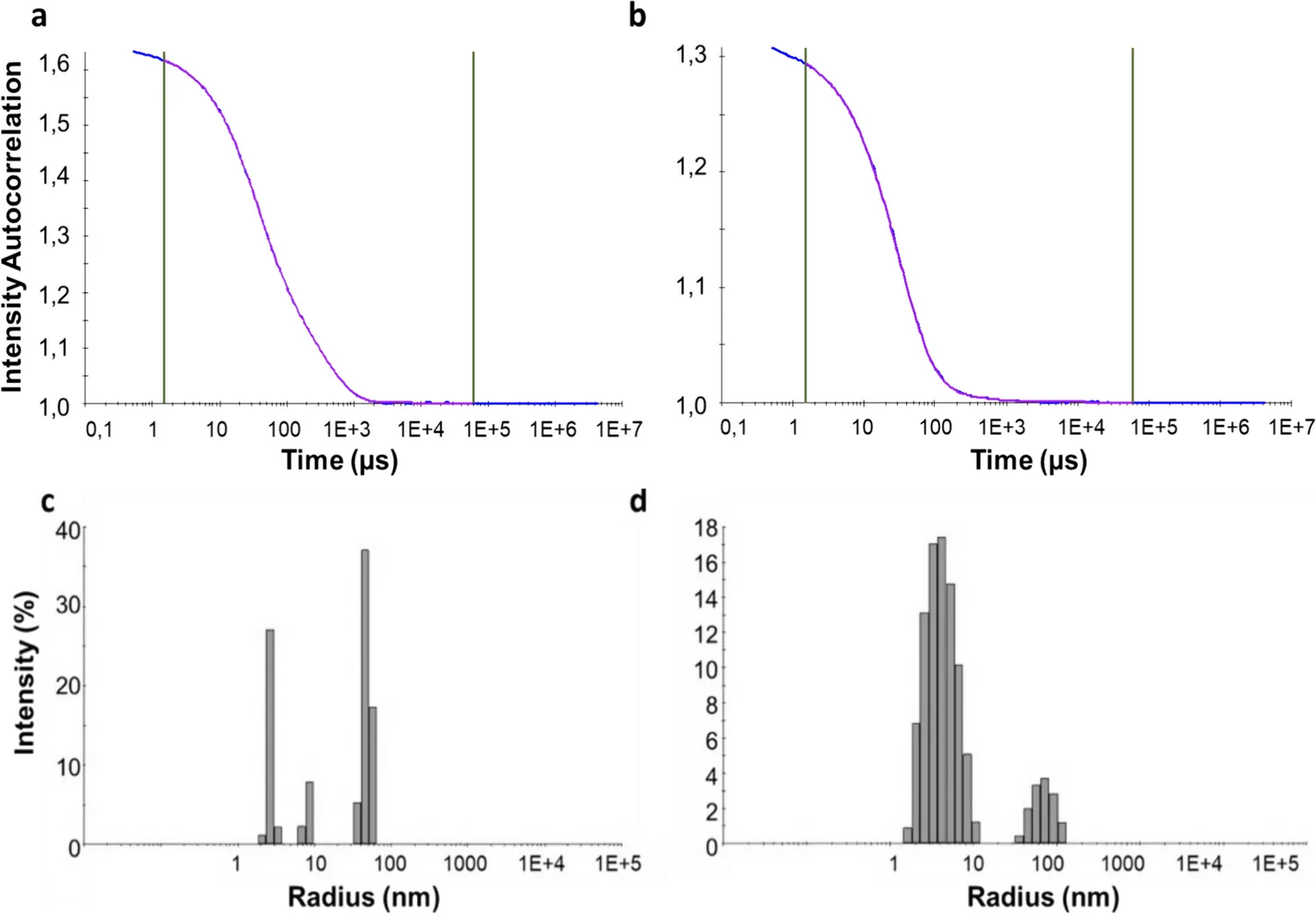
**a**, **b** Autocorrelation functions (ACF). Fitted models for multimodal sample (regularization or size distribution fit) (pink) and experimental data (blue) are represented .**c**, **d** Percentage of intensity as a function of the hydrodynamic radius (nm). Left – CspZ measurements. Right – FhbA measurements

Dynamic light scattering (DLS) was used to determine the size and size distribution of the populations of particles present in the purified protein solution. Indeed, aggregated forms and/or multimeric states of proteins cannot be detected on an SDS-PAGE, supporting the use of DLS to further assess the quality of the protein solutions. The autocorrelation function (ACF) is shown in Fig. 2a, b. In Fig. 2a, CspZ exhibits a good fit with the multimodal model, as does FhbA in Fig. 2b, while the cumulant fit applied for monomodal samples did not match at all to the experimental data (data not shown). This observation is further supported by a more detailed analysis of the data presented in Fig. 2c, d. Indeed, Fig. 2c indicates that CspZ solution displays a multimodal distribution, the peak corresponding to the protein with a hydrodynamic radius (R_H_) of 2.7 nm being monodisperse (PDI < 15%) with an estimated molecular mass, calculated based on the *R*_*H*_, of 34 kDa which matches well with the theoretical value (27 kDa). Two larger particle size peaks are also observed (*R*_*H*_ = 8.3 nm and R_H _= 48.7 nm) that may correspond to large aggregates or dust particles. However, these populations only represent 1% of the protein sample in terms of mass or number percentage; therefore, they are considered as negligible.

In contrast, the FhbA solution in Fig. 2d also presents a multimodal distribution but the peak corresponding to the protein (*R*_*H*_ = 4,4 nm) is highly polydisperse (PDI > 15%) suggesting that the sample is heterogenous with an estimated molar mass of 110.6 kDa which is much higher than the theoretical value (23 kDa). These observations suggest that this protein has the tendency to form multimers, with an averaged molar mass that matches with a tetramer. The large particle size peak (*R*_*H*_ = 71,7 nm) might correspond to big aggregates or dust particles, but they represent less than 1% of the mass or number percentage; therefore, they are also considered as negligible (Table 1).

**Table 1:** Parameters determined by DLS for each peak

Protein/Peak		Hydrodynamic Radius (nm)	% Polydispersity	Mw-*R* (kDa)	% Intensity	% Mass	% Number
CspZ	Peak 1	2.7	8.0	34.0	30.3	98.8	100.0
	Peak 2	8.3	9.3	475.6	10.1	1.2	0.0
	Peak 3	48.7	13.8	29829.8	59.6	0.1	0.0
FhbA	Peak 1	4.4	43.0	110.6	86.5	100.0	100.0
	Peak 2	71.7	30.1	73875.4	13.5	0.0	0.0

**Fig. 3:**
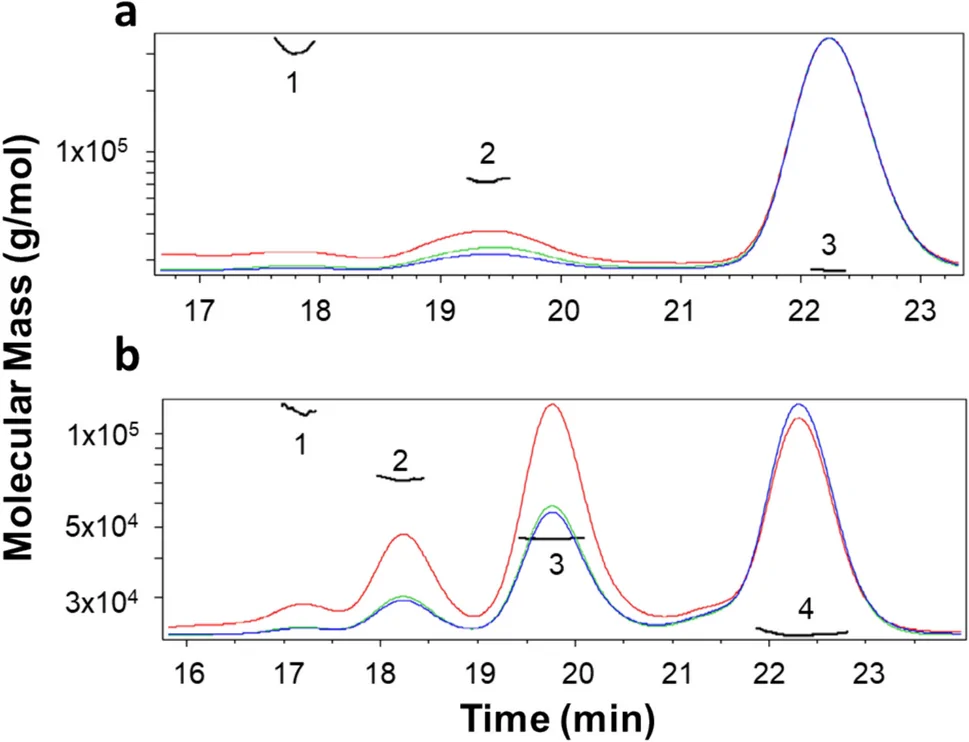
SEC-MALS analysis. Peaks and molecular masses are represented with numbers and black line. In red, light scattering; in green, UV; in blue, refractive index. **a** CspZ measurements. **b** FhbA measurements

To further analyze the oligomeric states of both protein solutions, SEC-MALS analysis was performed on both protein samples. The data presented in Fig. 3 and Table 2 show that CspZ is mainly monomeric with one major peak (91% of the protein sample) that exhibits a calculated molecular mass of 26.7 kDa which matches very well with the theoretical value (27.4 kDa). This monomeric form is highly homogenous (polydispersity = 1.000). A second peak is also detected with a calculated molar mass of 72.9 kDa which may correspond to a tetrameric form of the protein. However, this oligomeric state only represents 8% of the protein sample. Finally, a minor peak is also observed with a calculated molecular mass of 319.3 kDa. This form very likely corresponds to a soluble aggregated species but only represents 1% of the protein sample so it can be considered as negligible.

For FhbA, the elution profile is more complex, with more species that coexhist in the sample solution and are significantly populated. The monomeric form (calculated molecular mass of 22.9 kDa) represents 66% of the protein sample. Whereas the oligomeric forms of the protein: dimer; trimer and pentamer with calculated molecular masses of 46.2 kDa, 71.7 kDa, and 119.0 kDa represents 27%, 6%, and 1% of the protein sample. Each oligomeric form is monodisperse (maximum polydispersity of 1.001).

Altogether, this data confirm the propensy of FhbA to form oligomeric species in solution whereas CspZ is mainly monomeric in solution.

**Table 2:** Parameters determined by SEC-MALS for each peak

Protein/peak		Mw (kDa)	Polydispersity (Mw/Mn)	Representation (%)
CspZ	Peak 1	319.3	1.003	1
	Peak 2	72.9	1.000	8
	Peak 3	26.7	1.000	91
FhbA	Peak 1	119.0	1.001	1
	Peak 2	71.7	1.000	6
	Peak 3	46.2	1.000	27
	Peak 4	22.9	1.000	66

### Circular dichroism (CD) analysis

**Fig. 4:**
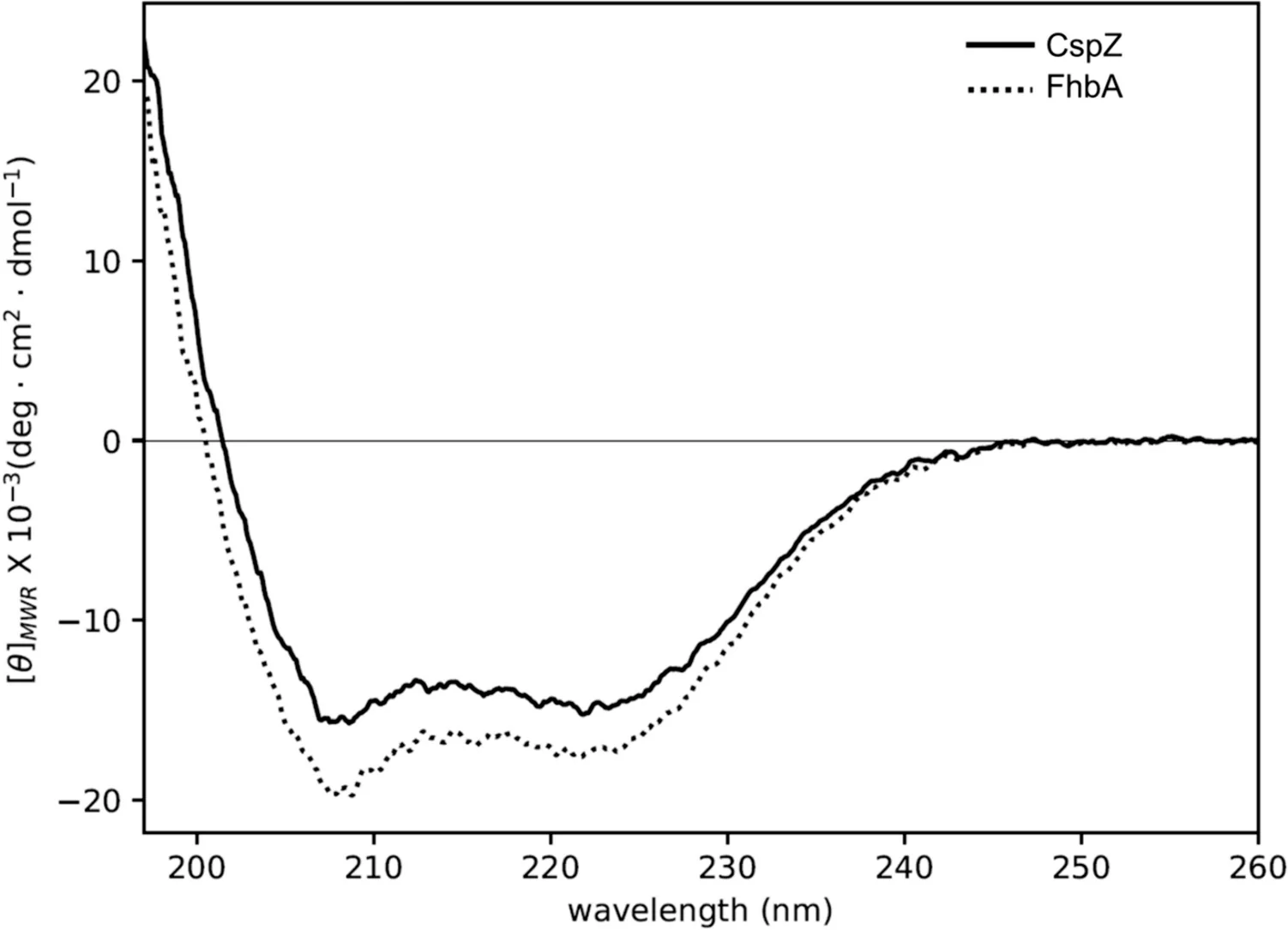
Far-UV CD spectra of (continuous line) CspZ and (dotted line) FhbA. Data were obtained at 20 °C, in PBS, with protein concentrations of ca. 0.1 mg/mL. Calculated helical contents from these spectra are (53.2 ± 1.4) % and (55.9 ± 1.3) %, respectively

Analysis of the far-UV CD spectrum (Fig. 4) of the two proteins unveiled a well-defined secondary structure organization. Both spectra exhibit two ellipticity minima around 222 and 208 nm, along with a strong positive signal below 200 nm, which are distinct signatures of native proteins with a high content of α-helical structures. Decomposition of the far-UV CD spectrum for both proteins revealed approximately 55% α-helices (refer to Table 3), a value lower than that derived from the analysis of the X-ray structures for each protein (approximately 72%).

**Table 3:** Calculated secondary structures derived from the far-UV CD data

Protein	Algorithm	Helix (%)	Strand (%)	Turn (%)	Unordered (%)	Total (%)
CspZ	CDSSTR	54.7	9.5	14.2	21.1	99.5
	CONTINLL	52.9	5.9	14.8	26.5	100.1
	SELCON3	51.9	8.1	14.6	24.8	99.4
	**Average**	**53.2 ± 1.4**	**7.8 ± 2.0**	**14.5 ± 0.3**	**24.1 ± 3.0**	**99.7**
FhbA	CDSSTR	57.4	6.2	12.6	23.7	99.9
	CONTINLL	55.3	4.1	11.7	29.0	100.0
	SELCON3	54.9	4.5	15.1	26.5	101.0
	**Average**	**55.9 ± 1.3**	**4.9 ± 1.0**	**13.1 ± 2.0**	**26.4 ± 3.0**	**100.3**

### NMR analysis

**Fig. 5:**
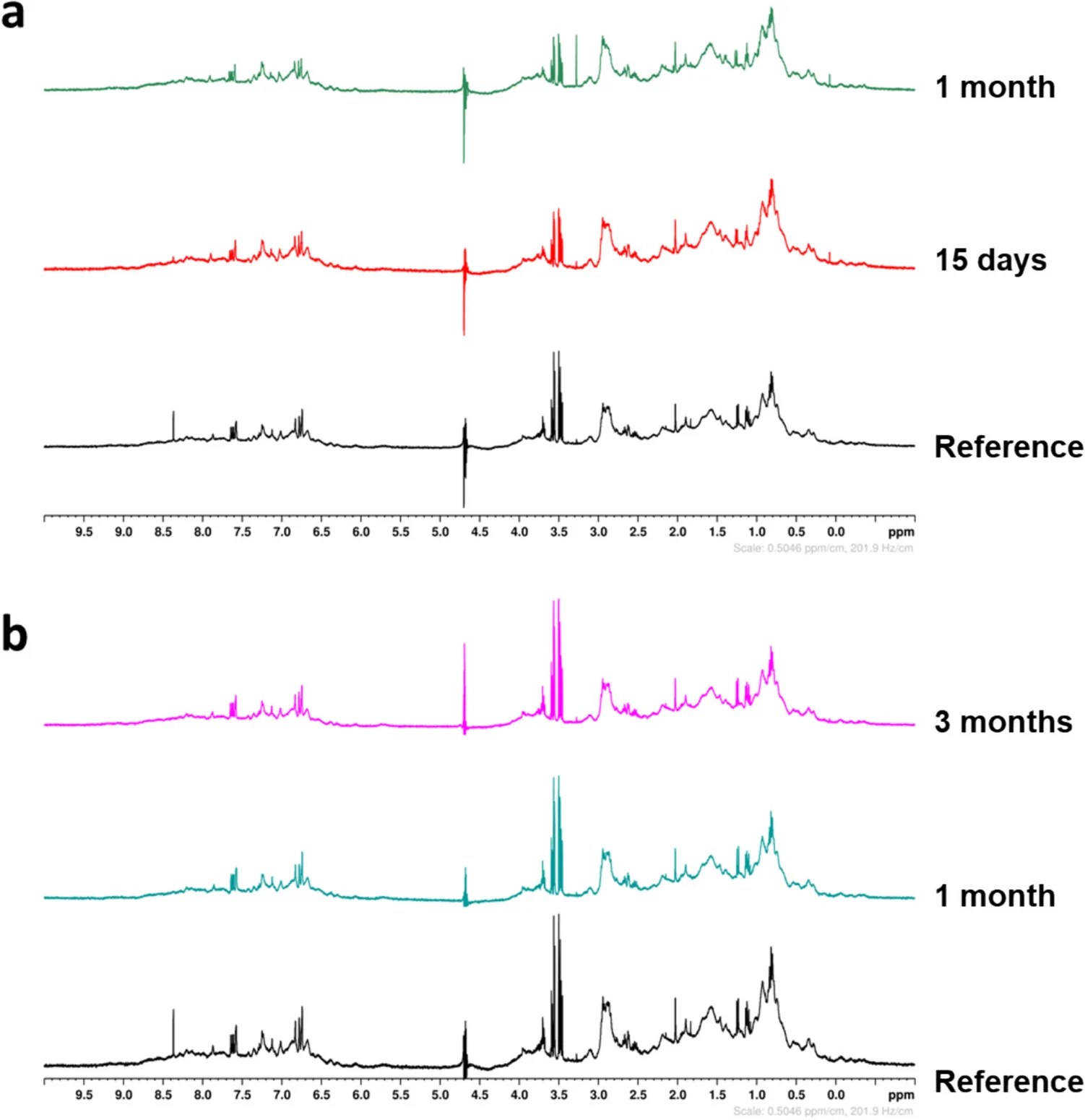
CspZ stability as a function of the storage conditions over time using one-dimensional 1 H NMR spectra approach. **a** Stability of CspZ at 4 °C at *t*_0_ (i.e. reference), 15 days and 1 month. **b** Stability of CspZ at −80 °C at* t*_0_ and after defrosting at 1 month and 3 months

NMR was performed only on CspZ to assess its stability when stored at 4 or −80 °C. Figure 5 shows no significant chemical shift change in the amide protons. Moreover, all spectra display the same narrow resonances with sharp lines, indicating the good stability of the protein at both 4 °C and −80 °C, regardless of the duration of the test.

### SPR analysis

**Fig. 6:**
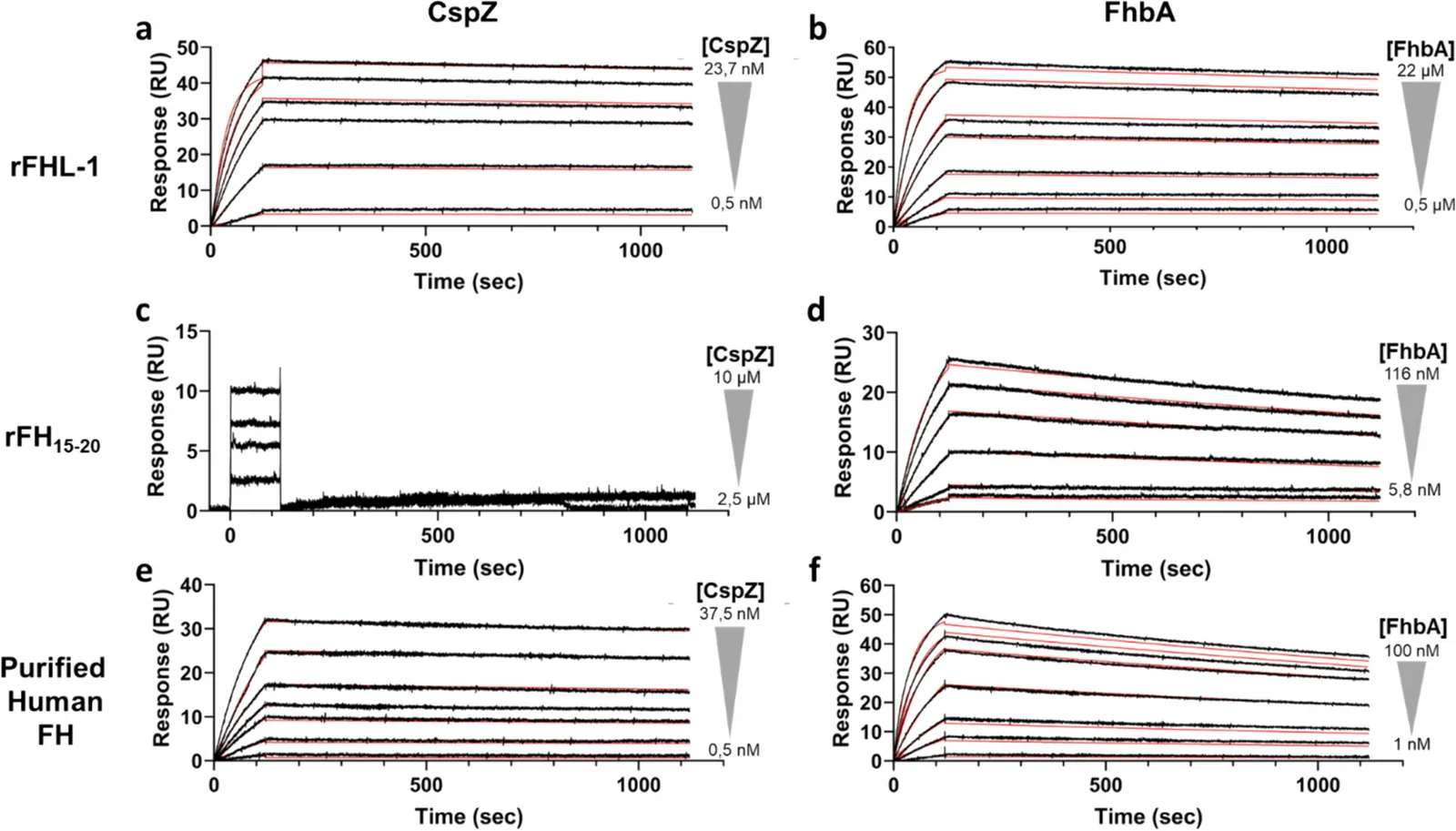
SPR sensorgrams of the interaction between FH, rFHL-1, or rFH_15-20_ and FhbA or CspZ. The ligand was immobilized on the surface of a CM5 sensor chip as described in the Materials and Methods section. Experimental sensorgrams are in black, and fitted sensorgrams are in red. **a** Analysis of the interaction between CspZ and immobilized rFHL-1. The interaction between CspZ and immobilized rFHL-1 was analyzed by injecting CspZ samples at the following concentrations: 0.5, 2.8, 6.6, 9.5, 14.2, and 23.7 nM. **b** Analysis of the interaction between FhbA and immobilized rFHL-1. FhbA samples were injected at 0.5, 1.1, 2.2, 4.5, 6.6, 13.4, and 22 µM. **c** Analysis of the interaction between CspZ and immobilized rFH_15-20_. CspZ samples were injected at 2.5, 5, 7.5, and, 10 µM. **d** Analysis of the interaction between FhbA and immobilized rFH_15-20_. FhbA samples were injected at 5.8, 11.6, 29, 58, 87, and 116 nM. **e** Analysis of the interaction between CspZ and immobilized FH. CspZ samples were injected at 0.5, 3, 7, 10, 15, 25, and 37.5 nM. **f** Analysis of the interaction between FhbA and immobilized FH. FhbA samples were injected at 1, 5, 10, 25, 50, 75, and 100 nM

The biological activity of the previously produced and purified proteins was assessed using surface plasmon resonance (SPR) analysis to determine the binding kinetics and affinities with FH, rFH_15 − 20_, and rFHL-1. Figure 6 presents the experimental data and sensorgrams. Table 4 summarizes the kinetic association and dissociation constants (*k*_on_ and *k*_off_) and the calculated equilibrium dissociation constant (K_D_). The results of the quantitative analysis indicate that CspZ has significantly higher affinities for FH and rFHL-1, with an equilibrium dissociation constant of 209 pM and 44 pM, respectively. Whereas for FhbA, the determined dissociation constants are 1.09 nM for FH and 42.95 nM for rFHL-1. Concerning FH, it can be observed that the difference in binding affinity is due to a more stable complex formed between FH and CspZ (lower *k*_off_) as compared to FhbA. Concerning rFHL-1, the difference in affinity seems to be mostly due to a much faster association kinetic between CspZ and FHL-1 (higher *k*_on_) as compared to FhbA. Furthermore, it is also worth mentioning that the interaction of FhbA is higher for rFH_15 − 20_ as compared to rFHL-1, indicating the importance of domain 20 of rFHL-1 for the binding affinity of FhbA. Conversely, in accordance with the data previously published (Hartmann et al. 2006; Kogan et al. 2022; Marcinkiewicz et al. 2023), we confirm that CspZ does not interact with rFH_15 − 20_, while FhbA is able to interact with rFH_15 − 20_, displaying a K_D_ of 3.37 nM.

**Table 4:** Quantitative analysis of the interaction between immobilized FH, rFHL-1, or rFH_15-20_ and CspZ or FhbA proteins

Ligand	Analyte	*k*_*on*_ (1/Ms)	*k*_*off*_ (1/s)	*K*_*D*_ (nM)
FH	FhbA	(2.46 ± 0.15) × 10^5^	(2.68 ± 0.65) × 10^−4^	1.086 ± 0.198
	CspZ	(2.55 ± 0.97) × 10^5^	(5.31 ± 2.53) × 10^−5^	0.209 ± 0.141
rFHL-1	FhbA	(1.57 ± 0.04) × 10^3^	(6.74 ± 1.36) × 10^−5^	42.945 ± 7.488
	CspZ	(7.20 ± 1.54) × 10^5^	(3.10 ± 0.32) × 10^−5^	0.044 ± 0.005
rFH_15-20_	FhbA	(8.54 ± 3.79) × 10^4^	(2.66 ± 0.29) × 10^−4^	3.367 ± 1.158
	CspZ	No interaction observed		

All experiments were performed in duplicate. All *x*² values and *U*-values were below 2 and 9, respectively

## Discussion

In this study, we produced and characterized two proteins, i.e., CspZ and FhbA, which are involved in the escape mechanisms to the human immune system of the *Borrelia* bacteria. This work contributes to the understanding of escape mechanisms employed by *Borrelia*. Additionally, the interaction between these proteins and two components of the human complement system, FH and FHL-1, was investigated. To achieve these objectives, a wide range of analytical techniques was employed to evaluate protein purity (SDS-PAGE, WB, HPLC), mass and size homogeneity (mass spectroscopy, DLS, SEC-MALS), stability (NMR), and secondary structure (CD) in accordance to published guidelines (de Marco et al. 2021). Furthermore, to confirm the functionality of the produced and purified proteins, their interaction with their targets, i.e., FH, rFH_15 − 20_, and rFHL-1, was characterized using SPR.

The two proteins were expressed and purified in a soluble fraction of *E. coli* using a single-step purification method with cobalt affinity chromatography for His-tagged proteins. The resulting solutions contained between 1 and 6 mg/mL of purified proteins. The HPLC analyses showed that CspZ is pure at > 99%, while the purity of FhbA was assessed at approximately 92%. The size distribution, measured by DLS, was presented using the percentage of intensity instead of the percentage of mass or number. This approach is preferred as it avoids the assumption that all particles have identical shapes and optical properties, which is not accurate. The DLS and SEC-MALS measurements indicate that the CspZ protein solution used in SPR experiments was of good quality and homogeneous, with no significant populations of aggregates. SEC-MALS experiments confirmed that only a very small fraction of CspZ sample was shown to form dimer. However, the FhbA protein solution exhibited polydispersity by DLS, with evidence of co-existing multimeric species in the solution. This observation was corroborated by mass spectrometry, which revealed a subpopulation corresponding to dimerized proteins, and by SEC-MALS data that detected the presence of dimer, trimer and pentamer in the protein solution. This multimerization could be an intrinsic property of the protein or a consequence of the formulation buffer used, which may not be optimal and might require further optimization. Additionally, the multimerization phenomenon is likely dependent on protein concentration and is highly dynamic. Notably, the DLS measurements were performed at a relatively high protein concentration (1 mg/mL), which could contribute to the formation of multimers. Altogether, SPR assays showed that FhbA was able to interact with FH, rFHL-1, and FH_15 − 20_ with high affinities comparable to published data (Table 5). This suggests that the multimeric species remain functionally active, with the ability to interact with their biological partners.

**Table 5:** Summary of interaction characterizations presented in this study, compared with available data from previous studies

Protein-protein interactions		K_D_ (nM) by SPR in this study	Published data, methodology, (reference)
CspZ	Human purified FH	0.21	+, WB, (Hartmann et al. 2006)
	rFH_15-20_	No interaction	No interaction, WB, (Hartmann et al. 2006)
	rFH_6-7_	*nd*	150 nM, SPR, (Marcinkiewicz et al. 2023) 290-310 nM, ELISA (Marcinkiewicz et al. 2023)
	rFHL-1	0.04	++, WB, (Hartmann et al. 2006)
FhbA	Human purified FH	1.09	30 nM, MST, (Kogan et al. 2022)
	rFH_19-20_	*nd*	82 nM, MST, (Kogan et al. 2022)
	rFH_15-20_	3.37	*np*
	rFHL-1	42.95	*np*

The symbols “+” or “++” indicate the level of interaction observed through WB analysis, while “nd” denotes “not determined” and “np” indicates “not published”

Previous studies employed CD spectroscopy to analyze protein folding (Marcinkiewicz et al. 2019; Kogan et al. 2022). Analysis of the two far-UV CD spectra indicates that approximately 50% of the residues in each protein are folded into α-helices. This value is significantly smaller than that obtained based on the crystal structures of the two proteins (approximately 72% α-helices; PDB: 6ATG and 6ZH1 for CspZ and FhbA, respectively). Although the possibility of significant conformational differences between the solution and crystalline states of these proteins cannot be ruled out, an underestimation of their solution concentrations is likely the cause of the underestimation of their α-helix content. Nonetheless, the estimation of the secondary structure content of a protein from its CD spectrum is empirical, primarily due to the absence of a unique solution for decomposing the spectrum and the various assumptions made in the process (Venyaminov and Yang 1996). Specifically, this analysis hinges on the assumption that only peptide chromophores contribute to the far-UV CD spectrum, while contributions from other chromophores within the protein are typically disregarded (Venyaminov and Yang 1996). Taken together, these considerations can explain the discrepancy in helical percentages observed between crystal structure and solution CD. Most importantly, our results indicate that the purified CspZ and FhbA proteins have acquired a well-defined secondary structure and are likely correctly folded. A stability study using NMR was conducted on CspZ. The comparison of the spectra obtained at different temperatures and times to the reference revealed no substantial chemical shift change of the amide protons, indicating that the local environment of the protons remained relatively consistent. The data indicate that CspZ is highly stable at 4 °C for up to 1 month, and can also be stored at −80 °C for an extended period of 3 months without compromising its structural integrity. These results suggest that CspZ can be stored for extended periods without any negative impact on its overall structure.

The success of subsequent interaction studies relies on a high purity and satisfactory protein quality of the purified proteins. To fully understand the modalities for the interaction of binding partners, it is essential to characterize the dissociation constant, in conjunction with the crystal structures between (i) CspZ with the domains SCR6-7 of FH/FHL-1 (PDB codes: 7ZJM and 6ATG) and (ii) FhbA with the domains SCR19-20 of FH (PDB code: 6ZH1). This objective was achieved using SPR. This showed strong binding interactions between CspZ and FhbA with both purified human FH and rFHL-1. Strong binding was also observed between rFH_15 − 20_ and FhbA, while no interaction was detected between rFH_15 − 20_ and CspZ. Table 5 summarizes the dissociation constant of each partner (when determined) and compares them with available literature data.

The symbols “+” or “++” indicate the level of interaction observed through WB analysis, while “nd” denotes “not determined” and “np” indicates “not published”.

For what concerns CspZ, SPR analysis showed a high affinity for FH (210 pM) and an even higher affinity for rFHL-1 (40 pM). As Kraiczy and collaborators previously noted (Kraiczy et al. 2004; Hartmann et al. 2006), the difference in affinity for CspZ may be due to the absence of a hydrophobic tail in FH and the smaller protein size of FHL-1. Marcinkiewicz and collaborators emphasized the importance of SCR6 and SCR7 for the interaction using SPR experiments (Marcinkiewicz et al. 2023). Additionally, Brangulis and collaborators demonstrated the key role of SCR7 using an X-ray crystallography approach (Brangulis et al. 2014). Overall, previous studies highlight the importance of the SCR7 in the bacterial competitive strategy within the human system. On the contrary, Hartmann et al. suggest that SCR15 to 20 are less essential for the interaction (Hartmann et al. 2006), a conclusion that is supported by our results (Table 5).

In relation to FhbA, its affinity is slightly weaker than that of CspZ, and the affinity trend is reversed. Indeed, the affinity for FH (1.09 nM) is higher than that for rFHL-1 (42.95 nM). These findings indicate the central role of the SCR20 domain for the interaction, as proposed by Kogan and colleagues (Kogan et al. 2022). This conclusion is supported by the observation that the interaction between FhbA and rFH_15 − 20_ is significantly stronger than the interaction between FhbA and rFHL-1, with a *K*_*D*_ of 3.37 nM compared to 42.95 nM, respectively. There are discrepancies in the interaction strength between FhbA and FH when comparing the values presented in Table 5. Specifically, the *K*_*D*_ determined in this study is approximately 30 times lower than that reported by Kogan and collaborators. This difference might depend from the distinct experimental techniques employed, namely SPR and MST, which involve immobilized and free protein, respectively. Our results reaffirm and complete the previous data for both proteins, validating the functionality of the expressed and purified recombinant proteins (Hartmann et al. 2006; Kraiczy et al. 2008a; Marcinkiewicz et al. 2019, 2020; Kogan et al. 2022).

In summary, this study provides a comprehensive understanding of the interaction between CspZ and FhbA, with FH and rFHL-1 by analyzing and determining the binding constants. This study characterizes and confirms the interaction of two outer surface proteins (CspZ and FhbA) from *B. burgdorferi* and *B. hermsii* respectively, which bind to human complement regulators, FH and FHL-1. The SPR analysis revealed a strong interaction in the range of pM to nM, which highlights the crucial role of these proteins in resisting the complement attack. Antibodies or other molecules that bind specifically to this interface could be utilized to design inhibitors that disrupt the interaction between *Borrelia* proteins and FH/FHL-1. Such inhibitors could potentially compromise *Borrelia’s* resistance to complement attack, leading to new therapeutic strategies. Finally, the lack of a direct and reliable detection method for LB remains a challenge. To overcome this issue, it is essential to produce and characterize functional bacterial surface proteins, since these proteins can be targeted for novel molecular probes, enabling the development of improved direct diagnostic tools.

## Data Availability

Data are available from the corresponding author.
